# Hypertension and Cognitive Decline: Implications of Obstructive Sleep Apnea

**DOI:** 10.3389/fcvm.2019.00096

**Published:** 2019-07-10

**Authors:** Meghna P. Mansukhani, Bhanu Prakash Kolla, Virend K. Somers

**Affiliations:** ^1^Center for Sleep Medicine, Mayo Clinic, Rochester, MN, United States; ^2^Department of Psychiatry and Psychology, Mayo Clinic, Rochester, MN, United States; ^3^Department of Cardiovascular Diseases, Mayo Clinic, Rochester, MN, United States

**Keywords:** blood pressure, dementia, sleep apnea, sleep deprivation, insufficient sleep, insomnia, sleepiness, somnolence

## Abstract

Hypertension and dementia are highly prevalent in the general population. Hypertension has been shown to be a risk factor for Alzheimer's dementia and vascular dementia. Sleep apnea, another common disorder, is strongly associated with hypertension and recent evidence suggests that it may also be linked with cognitive decline and dementia. It is possible that sleep apnea is the final common pathway linking hypertension to the development of dementia. This hypothesis merits further exploration as sleep apnea is readily treatable and such therapy could foreseeably delay or prevent the onset of dementia. At present, there is a paucity of therapeutic modalities that can prevent or arrest cognitive decline. In this review, we describe the associations between hypertension, dementia and sleep apnea, the pathophysiologic mechanisms underlying these associations, and the literature examining the impact of treatment of hypertension and sleep apnea on cognition. Potential areas of future investigation that may help advance our understanding of the magnitude and direction of the interaction between these conditions and the effects of treatment of high blood pressure and sleep apnea on cognition are highlighted.

## Introduction

Hypertension has recently been recognized as a risk factor for cognitive decline/dementia (1, 2). Both hypertension and cognitive decline/dementia are commonly seen in the general population (3, 4). A significant number of individuals with hypertension remain untreated, likely increasing their risk of long-term negative health consequences (3). Alzheimer's disease (AD) and vascular dementia account for the majority of the cases of dementia and most individuals with dementia have a combination of these two pathologies (5, 6). AD is the most common cause of dementia; it is a neurodegenerative disease, generally of older age, that is associated with excessive deposition of neurofibrillary tangles and amyloid plaques (7, 8). Vascular dementia is the second most common type of dementia accounting for 25–50% of all cases and refers to dementia in which cerebrovascular or cardiovascular disease is a primary cause or contributing factor (6, 9). Cardiovascular diseases, including hypertension, are risk factors for the development of AD as well as vascular dementia (6, 10). Additionally, cardiovascular diseases, AD and vascular dementia are thought to have shared genetic underpinnings and pathophysiology (11, 12).

Sleep disordered breathing or sleep apnea is also highly prevalent in the general population and is associated with several adverse cardiovascular outcomes (13–17). In its most common form, namely, obstructive sleep apnea (OSA), partial, or complete upper airway closure occurs during sleep in a repetitive fashion leading to hypoxemia and sleep fragmentation (18). OSA has been linked to incident and prevalent hypertension in several studies and it is thought that sympathetic overactivity leads to the development of high blood pressure (BP) in these individuals (19, 20). A growing body of evidence suggests that OSA is associated with cognitive impairment and dementia (21). It is possible that OSA plays a contributory role in the increased risk of cognitive decline and dementia seen in subjects with hypertension.

In this brief review, we describe the associations between hypertension and cognitive decline/dementia, between sleep apnea and hypertension, as well as between sleep apnea and cognitive decline/dementia. We discuss the putative pathophysiologic mechanisms underlying these associations. We also examine the literature assessing the effects of treatment of hypertension on cognitive function and the treatment of sleep apnea on BP and cognition. Last, we highlight areas of future investigation that may improve our understanding of the relationship between hypertension and sleep apnea as it pertains to the risk of cognitive decline/dementia, and the potential clinical impact of treatment of these conditions on the prevention of onset or worsening of cognitive decline.

## Hypertension and Cognitive Decline/Dementia

### Epidemiology

Hypertension has been shown to be associated with cognitive decline, AD and vascular dementia (1, 2). Several large epidemiologic studies have demonstrated a link between high BP in midlife (4th−5th decades of life) and cognitive decline or AD in later life, i.e., in the subsequent 20–30 years (19–23). This association has been more consistently established for diastolic BP (DBP) than systolic BP (SBP) (22, 23). A few investigations specifically assessing the relationship between midlife hypertension and vascular dementia have also found a significant association (4, 23). Supplementary Table S1 shows a summary of the epidemiologic studies examining the association between midlife elevated BP and cognitive decline/dementia.

The duration of high BP and the trajectory of BP over time appear to significantly influence the risk of cognitive decline/dementia (24, 25). Early-onset hypertension in childhood, adolescence or young adulthood, and duration of high SBP exceeding 25–30 years significantly enhances the risk, as does a combination of elevated BP in midlife coupled with low DBP in late-life (24–26). This is especially concerning, given that the prevalence of hypertension in younger individuals has been increasing steadily in the general population (27). Increased 24-h mean BP, 24-h variability of BP and non-dipping BP at night have all been associated with increased risk of cognitive impairment in those with and without hypertension (28–30). Interestingly, non-dipping of BP is extremely common in individuals with OSA and is discussed in more detail in the next section.

The association between late-life high BP and incident cognitive decline/dementia, both AD, and vascular dementia, is not consistent across studies (1). Hypertension in the 6th−7th decades of life has been linked with cognitive decline and a diagnosis of mild cognitive impairment (MCI) (31, 32). However, hypertension in the 8th−10th decades has not been found to increase such risk, and there are some data to suggest that it may in fact be protective against the development of cognitive impairment, especially in those with pre-existing chronic hypertension (33–35). This may in part reflect selection bias, in that those who do not develop cognitive impairment after many years are in a sense “resistant” to it. It is notable that in elderly individuals, low BP, very high SBP of >160 mm Hg and increased blood pressure variability have all been linked to cognitive impairment (36–39).

### Mechanisms

**Figure 1:**
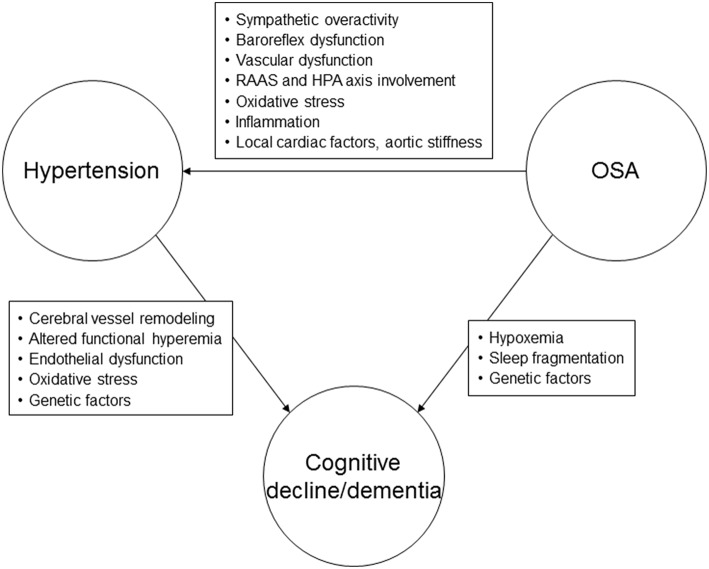
Possible pathophysiological mechanisms linking hypertension, obstructive sleep apnea, and cognitive decline or dementia. RAAS, renin-angiotensin-aldosterone system; HPA, hypothalamic-pituitary axis; OSA, obstructive sleep apnea.

Our understanding regarding the mechanisms underlying cognitive dysfunction in hypertension has recently been enhanced by advances in neuroimaging, which have consistently demonstrated effects of hypertension on brain structure and function. Increased SBP has been associated with reduced regional and total brain volumes, with further reductions in volume noted over time (40–42). The association between elevated DBP and brain volumes is not as clear (41–43). One of the main mechanisms underlying cognitive dysfunction in hypertension appears to involve cerebral vessel remodeling, leading to changes in cerebral autoregulation and perfusion, and thus affecting the ability to clear beta-amyloid (44) (Figure 1). Altered functional hyperemia, endothelial dysfunction and oxidative stress are other mechanisms that may play a contributory role (1). Subjects with hypertension demonstrate more beta-amyloid plaques, neurofibrillary tangles and cerebral atrophy compared to normotensive individuals (45–47). Subjects with hypertension also have reduced glucose metabolism in specific regions of the brain (47). Genetic variants may influence the effects of high BP on cognitive function. It appears that individuals with abnormal beta-amyloid levels and high midlife BP are at significantly increased risk of AD in the future (48). Some investigations have demonstrated that hypertension is linked to the development of cognitive impairment in subjects with the Apolipoprotein **ε**4 (APOE4) allele (48, 49).

Hypertension has been noted to be associated with the hallmark features of vascular dementia on neuroimaging studies, with reduced white matter microstructural integrity noted even prior to the development of overt neuroimaging abnormalities (50–53). The cognitive domains most affected by hypertension appear to be executive functioning and information processing speed (54, 55). There is accumulating evidence to suggest that treatment of hypertension decreases white matter hyperintensity progression (42, 52, 56). However, the relationship between hypertension and brain health is still not fully understood and can be affected by many factors including age, duration and trajectory of hypertension, as well as the degree of efficacy of antihypertensive medication.

### Treatment Effects

#### Findings

Prior randomized controlled trials evaluating the effects of antihypertensive treatment on cognitive function have shown mixed results (2). There was no convincing evidence of reduction in risk of cognitive decline in two placebo-controlled randomized controlled trials evaluating the effects of renin-angiotensin system blockade on cognitive function in subjects at high risk of cardiovascular disease (57, 58). A Cochrane systematic review also found no evidence that lowering of BP in late life reduced the risk of cognitive decline/dementia in hypertensive subjects (58). In the SPRINT MIND study, treating ambulatory adults with hypertension to a goal SBP of <120 mmHg compared to <140 mm Hg did not result in significant decrease in the risk of probable dementia after a mean follow-up of close to 6 years (59). However, there was a statistically significant reduction in risk of MCI (hazard ratio, 0.81, 95% CI, 0.69–0.95) noted in this study. A recent study showed that race may moderate of the impact of BP control on cognitive function (60). Results of other studies examining the impact of more intensive control of hypertension on cognitive function/risk of dementia are conflicting (59, 61). There is also evidence that excessive lowering of nocturnal BP may itself contribute to changes that may predispose to risk of dementia (62, 63). Amongst antihypertensive medications, it appears that angiotensin receptor blockers (ARBs) may be superior to other agents in decreasing the risk of cognitive decline/dementia according to a recent meta-analysis; however, this may be related to the possible neuroprotective effect of ARBs themselves rather than due to a reduction in BP (64).

#### Limitations

It remains uncertain if treatment of hypertension can change the course of cognitive decline/dementia. Several studies examining the effects of antihypertensive treatment on cognitive function had short durations of follow up. Additionally, many control subjects in the studies included in the Cochrane systematic review received antihypertensive treatment (58). The SPRINT MIND study was terminated early and there were fewer than expected cases of dementia, hence the study may have been underpowered to detect this endpoint (59, 65). Finally, most previous studies used the mini-mental state examination which has significant limitations in assessing cognitive outcomes (66).

#### Future Directions

Future studies should account for the shortcomings listed above and consider utilizing dementia-specific biomarkers as early indicators of cognitive decline and/or treatment-related effects, although is noteworthy that the use of biomarkers for this purpose has been debated in the literature (1, 67). Future investigations should consider the use of more comprehensive neurocognitive batteries for testing (66). The use of alternate markers of vessel function e.g., pulse pressure may provide further insights into the mechanisms underlying risk of cognitive decline/dementia in those with hypertension (1, 68, 69). The effects of varying degrees of BP control and the threshold below which BP needs to be lowered in order to improve neurocognitive outcomes needs clarification. Finally, the impact of BP reduction with antihypertensive medication on cognitive decline/dementia in various age, sex, ethnic, genetic, and clinical subgroups with hypertension requires further study (38, 69, 70).

## Sleep Apnea and Hypertension

### Epidemiology

Sleep apnea and hypertension are closely linked conditions, with shared risk factors such as age, male sex, and obesity (71). OSA is estimated to affect up to 24% of adult males and 9% of adult females in the United States (13). About half of subjects with hypertension have coexisting OSA and conversely, half of those with OSA have hypertension (72, 73).

Rapid eye movement (REM)-related OSA, a type of OSA in which disordered breathing events are mainly seen in REM sleep, has been independently associated with incident and prevalent hypertension (74, 75). Additionally, up to 80% of individuals with OSA demonstrate a non-dipping BP pattern at night; i.e., their BP does not demonstrate the normal physiological drop that occurs during sleep (76–78). Non-dipping BP status is a negative prognostic indicator, associated with increased risk of microalbuminuria, left ventricular hypertrophy, and cardiovascular morbidity and mortality. Very short-term (beat-by-beat) variability of BP is also related to severity of OSA, may be a negative cardiovascular prognostic indicator and amenable to treatment of OSA (79). Several recent studies have focused on the relationship between OSA and resistant hypertension (80–82). Approximately 70% of subjects with resistant hypertension have underlying OSA (80, 81). The increased risk of resistant hypertension in OSA appears to be particularly prominent in African-Americans, in whom undiagnosed OSA tends to be a highly prevalent condition (83, 84).

### Mechanisms

The mechanisms underlying hypertension in sleep apnea are thought to predominantly involve the chemoreflex response to hypoxemia (81, 85) (Figure 1). Impaired baroreflex responses, vascular dysfunction, systemic inflammation, and renin-angiotensin-aldosterone system involvement are other pathophysiologic mechanisms that may lead to elevated BP in subjects with OSA (81).

### Treatment Effects

#### Findings

The impact of treatment of OSA in lowering BP is modest, with a reduction in mean 24-h SBP and DBP of approximately 2–4 mm Hg (20, 86). The BP lowering effects of CPAP are noted in subjects with pre-hypertension and hypertension, and appear to be greater in those with a higher number of hours of device usage and those who are sleepy, comorbid diabetes mellitus, and possibly in those using fixed vs. auto-titrating modes of PAP (87, 88). Studies have suggested that CPAP treatment may reverse non-dipping of BP at night in those with resistant hypertension (89, 90). The magnitude of reduction in BP with CPAP therapy for OSA appears to be greater in those with resistant hypertension, in the range of 5–7 mm Hg for SBP and 3–6 mmHg for DBP (90–92). A recent study demonstrated that a cluster of micro-ribonucleic acids predicted a reduction in BP with CPAP (93). Another recent review described the potential role of mineralocorticoid antagonists in the treatment of resistant hypertension and decreasing the severity of SDB in subjects with OSA (94). Renal denervation has also been shown to decrease the severity of sleep apnea in subjects with resistant hypertension in a recent proof-of-concept randomized controlled trial (95).

#### Limitations

Studies evaluating the effects of treatment of OSA on BP have utilized varying methods of measurements of BP and have included differing populations with hypertension, some on antihypertensive medication(s), making it difficult to compare results across studies.

#### Future Directions

Further investigations are required to determine whether 24-h or beat-by beat variability in BP can lead to the development of cognitive decline/dementia in subjects with OSA, with or without coexisting hypertension, and whether treatment of OSA can ameliorate this risk. Future randomized controlled trials are required to assess the benefit of treatment of OSA in preventing the development of incident hypertension. Studies are also needed to clarify the comparative effects of various treatment modalities for sleep apnea on BP and the differential effects of treatment in various subpopulations of those with OSA, such as those with REM-related OSA, those with excessive daytime sleepiness, and in various ethnic subgroups.

## Sleep Apnea and Cognitive Decline/Dementia

### Epidemiology

There is a growing body of literature suggesting an association between sleep apnea and cognitive decline, particularly in elderly individuals (21, 96). Much of the evidence comes from population-based studies and there are some negative studies in this regard as well. As with the studies evaluating the relationship between sleep apnea and hypertension, varying methods of assessing sleep apnea and cognitive function utilized in the different studies render it challenging to compare results and make definitive conclusions about the association between sleep apnea and cognitive decline/dementia.

A recent meta-analysis conducted by Leng and colleagues included cross-sectional and longitudinal population-based studies with a minimum of 200 participants each, mean age of 40 years or older, in whom cognitive dysfunction was assessed based on a clinical diagnosis of cognitive impairment or by using standard tests (21). Sleep apnea was considered to be present if the AHI was 15 events per h or greater. Fourteen studies including 4 million male and female subjects from 5 different countries showed that sleep apnea was associated with an increased risk of cognitive impairment. The risk ratio for cognitive impairment in those with sleep apnea from the pooled analysis of 6 longitudinal studies was 1.26 (95% CI 1.05–1.50) and from 5 longitudinal studies (after removing 1 study that introduced significant heterogeneity) was 1.35 (95% CI 1.11–1.65). Pooled analysis from 7 cross-sectional studies revealed that those with sleep apnea exhibited a small worsening of executive functioning (standard mean difference −0.05, 95% CI −0.09–0.00). There was no association noted between sleep apnea and global cognition (6 studies) or delayed memory (5 studies) in pooled analyses from the cross-sectional studies in which these outcomes were available. No evidence of publication bias was found by the authors. Similar findings regarding the effects of sleep apnea on specific cognitive domains have been noted in other meta-analyses of studies conducted in clinical populations (97, 98). A previous meta-analysis of case-control studies showed that individuals with AD were five times more likely to have sleep apnea than subjects of the same age who were cognitively intact (99).

### Mechanisms

The mechanisms underlying the association between sleep apnea and cognitive impairment are not fully understood at this time, but may include hypoxemia, sleep fragmentation, daytime sleepiness, amyloid, and tau protein neuropathology, oxidative stress, inflammation and metabolic dysfunction (99–104) (Figure 1). Both insufficient sleep (including insomnia) and excessive daytime sleepiness have been linked with dementia (96). From large population-based cohorts of subjects with OSA it appears that hypoxemia, in particular, the degree of nocturnal desaturation, may be more important as a possible causative factor for cognitive decline/dementia than sleep fragmentation (105, 106). Chronic intermittent hypoxemia may result in vascular dysfunction, neuronal damage and impairment of the blood-brain barrier, affecting synaptic plasticity (107). Multiple structural brain abnormalities have been described in subjects with OSA, including white matter hyperintensities, cerebral microbleeds, perivascular spaces, and gray matter changes in the form of reduced cortical thickness in the temporal areas that were found to be associated with decreased verbal encoding on neuropsychological testing (108–111).

Studies have indicated that the effects of sleep apnea on cognition are more pronounced in APOE4 gene carriers and it may be that the presence of this genotype confers an increased risk of cellular damage from oxidative stress and promotes neural inflammation (112, 113). OSA may lead to early changes in the biomarkers of AD, which are potentially modifiable (102, 114). These include a wide array of cerebrospinal fluid and blood biomarkers such as amyloid-β, tau proteins, inflammatory cytokines, acute-phase proteins, antioxidants, homocysteine, and clusterin (102, 115, 116).

### Treatment Effects

#### Findings

The effects of treatment of OSA on cognitive function are unclear at this time, with only slight, and inconsistent improvements noted in the various measures of neurocognitive function utilized in the studies that did show benefit following treatment of OSA (117–119). A recent pilot study demonstrated significant improvements in psychomotor/cognitive processing speed in subjects with MCI who were adherent to CPAP for a year compared to subjects with MCI and OSA who were not adherent to CPAP treatment (120). Both the CPAP-adherent and non-adherent group had similar rates of hypertension.

#### Limitations

Several of the above studies evaluating the effect of sleep apnea on cognition did not specifically examine BP as a contributory factor. The presence and contribution of underlying sleep disorders to the symptoms and risk of dementia have not been elucidated.

#### Future Directions

Further studies are required to explore mechanisms underlying cognitive dysfunction in sleep apnea; in particular, the influence of hypoxemia vs. sleep fragmentation and genotypes that might predispose individuals to the development of cognitive impairment/dementia. Large cohort studies are required to help identify a specific biomarker panel associating sleep apnea with the risk of dementia. Studies utilizing more comprehensive neurophysiologic test batteries to understand the effects of SDB on specific cognitive domains are needed. The outcomes need to be defined more clearly, i.e., the presence of mild cognitive impairment vs. dementia or cognitive decline, ascertained by changes in measures on serial testing, need to be classified as different outcomes. Analyses of neurocognitive outcomes by various age groups are required to help stratify risk, as well as those that account for the influence of potential confounders such as body mass index, as noted by Leng et al. (21). Also noted by the authors of this meta-analysis, studies using AHI as a continuous variable rather than as a dichotomized measure to assess for the risk of sleep apnea on cognition are needed; additionally, differing hypopnea definitions utilizing varying oxyhemoglobin and/or arousal criteria can yield very different AHI values; thus, standardization of criteria measuring the severity of sleep apnea are required when assessing its impact on cognitive outcomes.

## Conclusions

Hypertension, cognitive impairment and sleep apnea are common in the general population. Recent literature points to an association between hypertension and cognitive impairment/dementia. The link between sleep apnea and hypertension has been well described and the pathophysiologic mechanisms underlying this relationship have been studied in human subjects. Sleep apnea has also been identified as a risk factor for cognitive impairment and dementia, but the mechanisms underlying this relationship have not been completely delineated. It is conceivable that sleep apnea provides a pathway connecting hypertension to the onset of cognitive decline or dementia. Alternatively, the repetitive nocturnal hypoxemia associated with sleep apnea may conceivably serve to potentiate effects of hypertension on cognitive decline/dementia, and/or increased blood pressure may conceivably potentiate the effects of sleep apnea on risk of dementia. While sleep apnea may not fully account for the development of cognitive impairment or dementia in those with hypertension, it may serve to heighten the risk in vulnerable individuals. This hypothesis merits further exploration given that sleep apnea is an eminently treatable condition and the relative lack of interventions that are currently available for the prevention or treatment of cognitive decline/dementia. Identification of sleep apnea, particularly in elderly individuals, may help predict the risk of cognitive impairment. Based on the current evidence, perhaps those with moderate to severe SDB should be closely followed in the clinical setting for occurrence of cognitive dysfunction, with consideration given to full neuropsychometric testing for early detection of cognitive impairment. There is limited evidence that treatment of OSA may help cognition and the results of studies are conflicting. Future studies are urgently needed to evaluate whether treatment of sleep apnea can help reverse cognitive deficits and delay or prevent the onset of dementia.

## Author Contributions

MM and BK wrote the manuscript. VS provided critical review and made changes to the manuscript.

### Conflict of Interest Statement

MM is the principal investigator on a research grant funded by ResMed Foundation evaluating the effects of adaptive servoventilation treatment of central apnea syndromes on healthcare utilization that is not relevant to the current work. MM is the recipient of the Paul and Ruby Tsai and Family Fund Career Development Award at Mayo Clinic. VS is a Consultant for Respicardia, ResMed, U-Health, GlaxoSmithKline, Roche and Bayer. He is an investigator on the SERVE-HF Steering Committee and is working with Mayo Health Solutions and their industry partners on intellectual property related to sleep and cardiovascular disease. The Philips Respironics Foundation has provided a gift to Mayo Foundation. The remaining author declares that the research was conducted in the absence of any commercial or financial relationships that could be construed as a potential conflict of interest.

## References

[1] Walker KA Power MC Gottesman RF. Defining the relationship between hypertension, cognitive decline, and dementia: a review. Curr Hypertens Rep. (2017) 19:24. 10.1007/s11906-017-0724-3 28299725 PMC6164165

[2] Iadecola C Yaffe K Biller J Bratzke LC Faraci FM Gorelick PB. Impact of hypertension on cognitive function: a scientific statement from the american heart association. Hypertension. (2016) 68:e67–94. 10.1161/HYP.0000000000000053 27977393 PMC5361411

[3] Mills KT Bundy JD Kelly TN Reed JE Kearney PM Reynolds K. Global disparities of hypertension prevalence and control: a systematic analysis of population-based studies from 90 countries. Circulation. (2016) 134:441–50. 10.1161/CIRCULATIONAHA.115.018912 27502908 PMC4979614

[4] Kennelly SP Lawlor BA Kenny RA. Blood pressure and dementia - a comprehensive review. therapeutic advances in neurological disorders. (2009) 2:241–60. 10.1177/1756285609103483 21179532 PMC3002634

[5] Prince M Bryce R Albanese E Wimo A Ribeiro W Ferri CP The global prevalence of dementia: a systematic review and metaanalysis. Alzheimer's & dementia. J Alzheimer's Assoc. (2013) 9:63–75.e2. 10.1016/j.jalz.2012.11.007 23305823

[6] Gorelick PB Scuteri A Black SE Decarli C Greenberg SM Iadecola C. Vascular contributions to cognitive impairment and dementia: a statement for healthcare professionals from the american heart association/american stroke association. Stroke. (2011) 42:2672–713. 10.1161/STR.0b013e3182299496 21778438 PMC3778669

[7] Hansen LA Samuel W. Criteria for Alzheimer's disease and the nosology of dementia with Lewy bodies. Neurology. (1997) 48:126–32. 10.1212/WNL.48.1.126 9008507

[8] McKhann GM Knopman DS Chertkow H Hyman BT Jack CR Jr Kawas CH. The diagnosis of dementia due to Alzheimer's disease: recommendations from the national institute on aging-Alzheimer's association workgroups on diagnostic guidelines for Alzheimer's disease. Alzheimer's & dementia. J Alzheimer's Assoc. (2011) 7:263–9. 10.1016/j.jalz.2011.03.005 21514250 PMC3312024

[9] Neuropathology Group Medical Research Council Cognitive Function and Aging Study Pathological correlates of late-onset dementia in a multicentre, community-based population in England and Wales. neuropathology group of the medical research council cognitive function and ageing study (MRC CFAS). Lancet (London, England). (2001) 357:169–75. 10.1016/S0140-6736(00)03589-3 11213093

[10] Rosendorff C Beeri MS Silverman JM. Cardiovascular risk factors for Alzheimer's disease. Am J Geriatric Cardiol. (2007) 16:143–9. 10.1111/j.1076-7460.2007.06696.x 17483665

[11] Traylor M Adib-Samii P Harold D Dichgans M Williams J Lewis CM. Shared genetic contribution to Ischaemic stroke and Alzheimer's disease. Ann Neurol. (2016) 79:739–47. 10.1002/ana.24621 26913989 PMC4864940

[12] Sonnen JA Larson EB Crane PK Haneuse S Li G Schellenberg GD. Pathological correlates of dementia in a longitudinal, population-based sample of aging. Ann Neurol. (2007) 62:406–13. 10.1002/ana.21208 17879383

[13] Peppard PE Young T Barnet JH Palta M Hagen EW Hla KM. Increased prevalence of sleep-disordered breathing in adults. Am J Epidemiol. (2013) 177:1006–14. 10.1093/aje/kws342 23589584 PMC3639722

[14] Heinzer R Vat S Marques-Vidal P Marti-Soler H Andries D Tobback N. Prevalence of sleep-disordered breathing in the general population: the HypnoLaus study. Lancet Respirat Med. (2015) 3:310–8. 10.1016/S2213-2600(15)00043-0 25682233 PMC4404207

[15] Somers VK White DP Amin R Abraham WT Costa F Culebras A. Sleep apnea and cardiovascular disease: an American heart association/american college of cardiology foundation scientific statement from the american heart association council for high blood pressure research professional education committee, council on clinical cardiology, stroke council, and council on cardiovascular nursing. in collaboration with the national heart, lung, and blood institute national center on sleep disorders research (national institutes of health). Circulation. (2008) 118:1080–111. 10.1161/CIRCULATIONAHA.107.189420 18725495

[16] Bradley TD Floras JS. Obstructive sleep apnoea and its cardiovascular consequences. Lancet (London, England). (2009) 373:82–93. 10.1016/S0140-6736(08)61622-0 19101028

[17] Javaheri S Barbe F Campos-Rodriguez F Dempsey JA Khayat R Javaheri S. Sleep apnea: types, mechanisms, and clinical cardiovascular consequences. J Am Coll Cardiol. (2017) 69:841–58. 10.1016/j.jacc.2016.11.069 28209226 PMC5393905

[18] Kapur VK Auckley DH Chowdhuri S Kuhlmann DC Mehra R Ramar K. Clinical practice guideline for diagnostic testing for adult obstructive sleep apnea: an american academy of sleep medicine clinical practice guideline. J Clin Sleep Med. (2017) 13:479–504. 10.5664/jcsm.6506 28162150 PMC5337595

[19] Xia W Huang Y Peng B Zhang X Wu Q Sang Y. Relationship between obstructive sleep apnoea syndrome and essential hypertension: a dose-response meta-analysis. Sleep Med. (2018) 47:11–8. 10.1016/j.sleep.2018.03.016 29880142

[20] Mansukhani MP Kara T Caples SM Somers VK. Chemoreflexes, sleep apnea, and sympathetic dysregulation. Curr Hypertens Rep. (2014) 16:476. 10.1007/s11906-014-0476-2 25097113 PMC4249628

[21] Leng Y McEvoy CT Allen IE Yaffe K. Association of sleep-disordered breathing with cognitive function and risk of cognitive impairment: a systematic review and meta-analysis. JAMA Neurol. (2017) 74:1237–45. 10.1001/jamaneurol.2017.2180 28846764 PMC5710301

[22] Kilander L Nyman H Boberg M Lithell H. The association between low diastolic blood pressure in middle age and cognitive function in old age. a population-based study. Age Age. (2000) 29:243–8. 10.1093/ageing/29.3.243 10855907

[23] Launer LJ Ross GW Petrovitch H Masaki K Foley D White LR. Midlife blood pressure and dementia: the Honolulu-Asia aging study. Neurobiol Aging. (2000) 21:49–55. 10.1016/S0197-4580(00)00096-8 10794848

[24] Swan GE Carmelli D Larue A. Systolic blood pressure tracking over 25 to 30 years and cognitive performance in older adults. Stroke. (1998) 29:2334–40. 10.1161/01.STR.29.11.2334 9804644

[25] Power MC Tchetgen EJ Sparrow D Schwartz J Weisskopf MG. Blood pressure and cognition: factors that may account for their inconsistent association. Epidemiology (Cambridge, Mass). (2013) 24:886–93. 10.1097/EDE.0b013e3182a7121c 24030502 PMC3818218

[26] Glodzik L Rusinek H Pirraglia E McHugh P Tsui W Williams S. Blood pressure decrease correlates with tau pathology and memory decline in hypertensive elderly. Neurobiol Aging. (2014) 35:64–71. 10.1016/j.neurobiolaging.2013.06.011 23969178 PMC3799812

[27] McNiece KL Poffenbarger TS Turner JL Franco KD Sorof JM Portman RJ. Prevalence of hypertension and pre-hypertension among adolescents. J Pediatrics. (2007) 150:640–4, 4.e1. 10.1016/j.jpeds.2007.01.052 17517252

[28] Kanemaru A Kanemaru K Kuwajima I. The effects of short-term blood pressure variability and nighttime blood pressure levels on cognitive function. Hypertens Res. (2001) 24:19–24. 10.1291/hypres.24.19 11213025

[29] Bellelli G Frisoni GB Lucchi E Guerini F Geroldi C Magnifico F. Blunted reduction in night-time blood pressure is associated with cognitive deterioration in subjects with long-standing hypertension. Blood Pressure Monitor. (2004) 9:71–6. 10.1097/00126097-200404000-00003 15096903

[30] Sakakura K Ishikawa J Okuno M Shimada K Kario K. Exaggerated ambulatory blood pressure variability is associated with cognitive dysfunction in the very elderly and quality of life in the younger elderly. Am J Hypertens. (2007) 20:720–7. 10.1016/j.amjhyper.2007.02.001 17586405

[31] Elias MF Wolf PA D'Agostino RB Cobb J White LR. Untreated blood pressure level is inversely related to cognitive functioning: the framingham study. Am J Epidemiol. (1993) 138:353–64. 10.1093/oxfordjournals.aje.a116868 8213741

[32] Reitz C Tang MX Manly J Mayeux R Luchsinger JA. Hypertension and the risk of mild cognitive impairment. Arch Neurol. (2007) 64:1734–40. 10.1001/archneur.64.12.1734 18071036 PMC2672564

[33] Scherr PA Hebert LE Smith LA Evans DA. Relation of blood pressure to cognitive function in the elderly. Am J Epidemiol. (1991) 134:1303–15. 10.1093/oxfordjournals.aje.a116033 1755444

[34] Waldstein SR Giggey PP Thayer JF Zonderman AB. Nonlinear relations of blood pressure to cognitive function: the baltimore longitudinal study of aging. Hypertension. (2005) 45:374–9. 10.1161/01.HYP.0000156744.44218.74 15699446

[35] Corrada MM Hayden KM Paganini-Hill A Bullain SS DeMoss J Aguirre C Age of onset of hypertension and risk of dementia in the oldest-old: the 90+ study. Alzheimer's & dementia. J Alzheimer's Assoc. (2017) 13:103–10. 10.1016/j.jalz.2016.09.007 PMC5318224 28108119

[36] Glynn RJ Beckett LA Hebert LE Morris MC Scherr PA Evans DA. Current and remote blood pressure and cognitive decline. JAMA. (1999) 281:438–45. 10.1001/jama.281.5.438 9952204

[37] Bohannon AD Fillenbaum GG Pieper CF Hanlon JT Blazer DG. Relationship of race/ethnicity and blood pressure to change in cognitive function. J Am Geriatrics Soc. (2002) 50:424–9. 10.1046/j.1532-5415.2002.50104.x 11943035

[38] Zonneveld TP Richard E Vergouwen MD Nederkoorn PJ de Haan R Roos YB Blood pressure-lowering treatment for preventing recurrent stroke, major vascular events, and dementia in patients with a history of stroke or transient ischaemic attack. Cochrane Data System Rev. (2018) 7:Cd007858 10.1002/14651858.CD007858.pub2 PMC6513249 30024023

[39] Oishi E Ohara T Sakata S Fukuhara M Hata J Yoshida D. Day-to-day blood pressure variability and risk of dementia in a general japanese elderly population: the hisayama study. Circulation. (2017) 136:516–25. 10.1161/CIRCULATIONAHA.116.025667 28784822 PMC5548511

[40] Leritz EC Salat DH Williams VJ Schnyer DM Rudolph JL Lipsitz L. Thickness of the human cerebral cortex is associated with metrics of cerebrovascular health in a normative sample of community dwelling older adults. NeuroImage. (2011) 54:2659–71. 10.1016/j.neuroimage.2010.10.050 21035552 PMC3026290

[41] Nagai M Hoshide S Ishikawa J Shimada K Kario K. Ambulatory blood pressure as an independent determinant of brain atrophy and cognitive function in elderly hypertension. J Hypertens. (2008) 26:1636–41. 10.1097/HJH.0b013e3283018333 18622243

[42] Firbank MJ Wiseman RM Burton EJ Saxby BK O'Brien JT Ford GA. Brain atrophy and white matter hyperintensity change in older adults and relationship to blood pressure. brain atrophy, WMH change and blood pressure. J Neurol. (2007) 254:713–21. 10.1007/s00415-006-0238-4 17446997

[43] Harris P Alcantara DA Amenta N Lopez OL Eiriksdottir G Sigurdsson S. Localized measures of callosal atrophy are associated with late-life hypertension: AGES-reykjavik study. NeuroImage. (2008) 43:489–96. 10.1016/j.neuroimage.2008.07.007 18692143 PMC2590639

[44] Muller M van der Graaf Y Visseren FL Mali WP Geerlings MI. Hypertension and longitudinal changes in cerebral blood flow: the SMART-MR study. Anna Neurol. (2012) 71:825–33. 10.1002/ana.23554 22447734

[45] Rodrigue KM Rieck JR Kennedy KM Devous MD Sr. Diaz-Arrastia R Park DC. Risk factors for beta-amyloid deposition in healthy aging: vascular and genetic effects. JAMA Neurol. (2013) 70:600–6. 10.1001/jamaneurol.2013.1342 23553344 PMC3968915

[46] Petrovitch H White LR Izmirilian G Ross GW Havlik RJ Markesbery W. Midlife blood pressure and neuritic plaques, neurofibrillary tangles, and brain weight at death: the HAAS. honolulu-asia aging study. Neurobiol Aging. (2000) 21:57–62. 10.1016/S0197-4580(00)00106-8 10794849

[47] Langbaum JB Chen K Launer LJ Fleisher AS Lee W Liu X. Blood pressure is associated with higher brain amyloid burden and lower glucose metabolism in healthy late middle-age persons. Neurobiol Aging. (2012) 33:827.e11–9. 10.1016/j.neurobiolaging.2011.06.020 21821316 PMC3236809

[48] Andrews S Das D Anstey KJ Easteal S. Interactive effect of APOE genotype and blood pressure on cognitive decline: the PATH through life study. J Alzheimer's Dis. (2015) 44:1087–98. 10.3233/JAD-140630 25672766

[49] de Frias CM Schaie KW Willis SL. Hypertension moderates the effect of APOE on 21-year cognitive trajectories. Psychol Aging. (2014) 29:431–9. 10.1037/a0036828 24956008

[50] Raz N Rodrigue KM Kennedy KM Acker JD. Vascular health and longitudinal changes in brain and cognition in middle-aged and older adults. Neuropsychology. (2007) 21:149–57. 10.1037/0894-4105.21.2.149 17402815

[51] Gottesman RF Coresh J Catellier DJ Sharrett AR Rose KM Coker LH. Blood pressure and white-matter disease progression in a biethnic cohort: atherosclerosis risk in communities (ARIC) study. Stroke. (2010) 41:3–8. 10.1161/STROKEAHA.109.566992 19926835 PMC2803313

[52] Verhaaren BF Vernooij MW de Boer R Hofman A Niessen WJ van der Lugt A. High blood pressure and cerebral white matter lesion progression in the general population. Hypertension. (2013) 61:1354–9. 10.1161/HYPERTENSIONAHA.111.00430 23529163

[53] Poels MM Zaccai K Verwoert GC Vernooij MW Hofman A van der Lugt A. Arterial stiffness and cerebral small vessel disease: the rotterdam scan study. Stroke. (2012) 43:2637–42. 10.1161/STROKEAHA.111.642264 22879099

[54] Lande MB Batisky DL Kupferman JC Samuels J Hooper SR Falkner B. Neurocognitive function in children with primary hypertension. J Pediatrics. (2017) 180:148–55.e1. 10.1016/j.jpeds.2016.08.076 27692987 PMC5183510

[55] Muela HC Costa-Hong VA Yassuda MS Moraes NC Memoria CM Machado MF. Hypertension severity is associated with impaired cognitive performance. J Am Heart Assoc. (2017) 6:e004579. 10.1161/JAHA.116.004579 28077386 PMC5523638

[56] Dufouil C Chalmers J Coskun O Besancon V Bousser MG Guillon P. Effects of blood pressure lowering on cerebral white matter hyperintensities in patients with stroke: the PROGRESS (perindopril protection against recurrent stroke study) magnetic resonance imaging substudy. Circulation. (2005) 112:1644–50. 10.1161/CIRCULATIONAHA.104.501163 16145004

[57] McGuinness B Todd S Passmore P Bullock R Blood pressure lowering in patients without prior cerebrovascular disease for prevention of cognitive impairment and dementia. Cochrane Data System Rev. 2009:Cd004034 10.1002/14651858.CD004034.pub3 PMC7163274 19821318

[58] Anderson C Teo K Gao P Arima H Dans A Unger T. Renin-angiotensin system blockade and cognitive function in patients at high risk of cardiovascular disease: analysis of data from the ONTARGET and TRANSCEND studies. Lancet Neurol. (2011) 10:43–53. 10.1016/S1474-4422(10)70250-7 20980201

[59] Williamson JD Pajewski NM Auchus AP Bryan RN Chelune G Cheung AK. Effect of intensive vs standard blood pressure control on probable dementia: a randomized clinical trial. JAMA. (2019) 321:553–561. 10.1001/jama.2018.21442 30688979 PMC6439590

[60] Hajjar I Rosenberger KJ Kulshreshtha A Ayonayon HN Yaffe K Goldstein FC. Association of JNC-8 and SPRINT systolic blood pressure levels with cognitive function and related racial disparity. JAMA Neurol. (2017) 74:1199–205. 10.1001/jamaneurol.2017.1863 28828478 PMC5710237

[61] Lamar M Wu D Durazo-Arvizu RA Brickman AM Gonzalez HM Tarraf W. Cognitive associates of current and more intensive control of hypertension: findings from the hispanic community health study/study of latinos. Am J Hyper. (2017) 30:624–31. 10.1093/ajh/hpx023 28402388 PMC5861562

[62] Bohm M Schumacher H Teo KK Lonn EM Mahfoud F Mann JFE. Achieved blood pressure and cardiovascular outcomes in high-risk patients: results from ONTARGET and TRANSCEND trials. Lancet (London, England). (2017) 389:2226–37. 10.1016/S0140-6736(17)30754-7 28390695

[63] Vidal-Petiot E Ford I Greenlaw N Ferrari R Fox KM Tardif JC. Cardiovascular event rates and mortality according to achieved systolic and diastolic blood pressure in patients with stable coronary artery disease: an international cohort study. Lancet (London, England). (2016) 388:2142–52. 10.1016/S0140-6736(16)31326-5 27590221

[64] Levi Marpillat N Macquin-Mavier I Tropeano AI Bachoud-Levi AC Maison P. Antihypertensive classes, cognitive decline and incidence of dementia: a network meta-analysis. J Hypertens. (2013) 31:1073–82. 10.1097/HJH.0b013e3283603f53 23552124

[65] Yaffe K. Prevention of cognitive impairment with intensive systolic blood pressure control. JAMA. (2019) 321:548–9. 10.1001/jama.2019.0008 30688980

[66] Bossers WJ van der Woude LH Boersma F Scherder EJ van Heuvelen MJ. Recommended measures for the assessment of cognitive and physical performance in older patients with dementia: a systematic review. Dementia Geriatric Cognit Disorders Extra. (2012) 2:589–609. 10.1159/000345038 23341825 PMC3551396

[67] Hughes TM Sink KM. Hypertension and its role in cognitive function: current evidence and challenges for the future. Am J Hypertension. (2016) 29:149–57. 10.1093/ajh/hpv180 26563965 PMC4989128

[68] Nation DA Edmonds EC Bangen KJ Delano-Wood L Scanlon BK Han SD. Pulse pressure in relation to tau-mediated neurodegeneration, cerebral amyloidosis, and progression to dementia in very old adults. JAMA Neurol. (2015) 72:546–53. 10.1001/jamaneurol.2014.4477 25822631 PMC4428938

[69] Levine DA Galecki AT Langa KM Unverzagt FW Kabeto MU Giordani B. Blood pressure and cognitive decline over 8 years in middle-aged and older black and white americans. Hypertension. (2019) 73:310–8. 10.1161/HYPERTENSIONAHA.118.12062 30624986 PMC6450556

[70] Gilsanz P Mayeda ER Glymour MM Quesenberry CP Mungas DM DeCarli C. Female sex, early-onset hypertension, and risk of dementia. Neurology. (2017) 89:1886–93. 10.1212/WNL.0000000000004602 28978656 PMC5664296

[71] Min HJ Cho YJ Kim CH Kim DH Kim HY Choi JI. Clinical features of obstructive sleep apnea that determine its high prevalence in resistant hypertension. Yonsei Med J. (2015) 56:1258–65. 10.3349/ymj.2015.56.5.1258 26256968 PMC4541655

[72] Konecny T Kara T Somers VK. Obstructive sleep apnea and hypertension: an update. Hypertension. (2014) 63:203–9. 10.1161/HYPERTENSIONAHA.113.00613 24379177 PMC4249687

[73] Drager LF Genta PR Pedrosa RP Nerbass FB Gonzaga CC Krieger EM. Characteristics and predictors of obstructive sleep apnea in patients with systemic hypertension. Am J Cardiol. (2010) 105:1135–9. 10.1016/j.amjcard.2009.12.017 20381666

[74] Mokhlesi B Finn LA Hagen EW Young T Hla KM Van Cauter E. Obstructive sleep apnea during REM sleep and hypertension. results of the wisconsin sleep cohort. Am J Respir Crit Care Med. (2014) 190:1158–67. 10.1164/rccm.201406-1136OC 25295854 PMC4299639

[75] Acosta-Castro P Hirotsu C Marti-Soler H Marques-Vidal P Tobback N Andries D. REM-associated sleep apnoea: prevalence and clinical significance in the HypnoLaus cohort. Eur Respir J. (2018) 52:1702484. 10.1183/13993003.02484-2017 29976653

[76] Wolf J Hering D Narkiewicz K. Non-dipping pattern of hypertension and obstructive sleep apnea syndrome. Hypertens Res. (2010) 33:867–71. 10.1038/hr.2010.153 20818398

[77] Redon J Lurbe E. Nocturnal blood pressure versus nondipping pattern: what do they mean? Hypertension. (2008) 51:41–2. 10.1161/HYPERTENSIONAHA.107.101337 18071059

[78] Sasaki N Ozono R Edahiro Y Ishii K Seto A Okita T. Impact of non-dipping on cardiovascular outcomes in patients with obstructive sleep apnea syndrome. Clin Exp Hypertens. (2015) 37:449–53. 10.3109/10641963.2015.1057833 26395950

[79] Marrone O Riccobono L Salvaggio A Mirabella A Bonanno A Bonsignore MR. Catecholamines and blood pressure in obstructive sleep apnea syndrome. Chest. (1993) 103:722–7. 10.1378/chest.103.3.722 8449058

[80] Pedrosa RP Drager LF Gonzaga CC Sousa MG de Paula LK Amaro AC. Obstructive sleep apnea: the most common secondary cause of hypertension associated with resistant hypertension. Hypertension. (2011) 58:811–7. 10.1161/HYPERTENSIONAHA.111.179788 21968750

[81] Mansukhani MP Wang S Somers VK. Chemoreflex physiology and implications for sleep apnoea: insights from studies in humans. Exp Physiol. (2015) 100:130–5. 10.1113/expphysiol.2014.082826 25398715 PMC4439216

[82] Walia HK Li H Rueschman M Bhatt DL Patel SR Quan SF. Association of severe obstructive sleep apnea and elevated blood pressure despite antihypertensive medication use. J Clin Sleep Med. (2014) 10:835–43. 10.5664/jcsm.3946 25126027 PMC4106935

[83] Johnson DA Thomas SJ Abdalla M Guo N Yano Y Rueschman M Association between sleep apnea and blood pressure control among blacks: jackson heart sleep study. Circulation. (2018) 139:1275–84. 10.1161/CIRCULATIONAHA.118.036675 PMC6428682 30586763

[84] Johnson DA Guo N Rueschman M Wang R Wilson JG Redline S. Prevalence and correlates of obstructive sleep apnea among African Americans: the jackson heart sleep study. Sleep. (2018) 41:zsy154. 10.1093/sleep/zsy154 30192958 PMC6187109

[85] Mansukhani MP Wang S Somers VK. Sleep, death, and the heart. Am J Physiol Heart Circ Physiol. (2015) 309:H739–49. 10.1152/ajpheart.00285.2015 26188022 PMC4591406

[86] Ren R Li Y Zhang J Zhou J Sun Y Tan L. Obstructive sleep apnea with objective daytime sleepiness is associated with hypertension. Hypertension. (2016) 68:1264–70. 10.1161/HYPERTENSIONAHA.115.06941 27620392

[87] Barbe F Duran-Cantolla J Sanchez-de-la-Torre M Martinez-Alonso M Carmona C Barcelo A. Effect of continuous positive airway pressure on the incidence of hypertension and cardiovascular events in nonsleepy patients with obstructive sleep apnea: a randomized controlled trial. JAMA. (2012) 307:2161–8. 10.1001/jama.2012.4366 22618923

[88] Mansukhani MP Covassin N Somers VK. Apneic sleep, insufficient sleep, and hypertension. Hypertension. (2019) 73:744–56. 10.1161/HYPERTENSIONAHA.118.11780 30776972 PMC6513351

[89] Varounis C Katsi V Kallikazaros IE Tousoulis D Stefanadis C Parissis J. Effect of CPAP on blood pressure in patients with obstructive sleep apnea and resistant hypertension: a systematic review and meta-analysis. Int J Cardiol. (2014) 175:195–8. 10.1016/j.ijcard.2014.04.240 24841834

[90] Martinez-Garcia MA Capote F Campos-Rodriguez F Lloberes P Diaz de Atauri MJ Somoza M. Effect of CPAP on blood pressure in patients with obstructive sleep apnea and resistant hypertension: the HIPARCO randomized clinical trial. JAMA. (2013) 310:2407–15. 10.1001/jama.2013.281250 24327037

[91] Iftikhar IH Valentine CW Bittencourt LR Cohen DL Fedson AC Gislason T. Effects of continuous positive airway pressure on blood pressure in patients with resistant hypertension and obstructive sleep apnea: a meta-analysis. J Hypertens. (2014) 32:2341–50; discussion 50. 10.1097/HJH.0000000000000372 25243523 PMC4291165

[92] Liu L Cao Q Guo Z Dai Q. Continuous positive airway pressure in patients with obstructive sleep apnea and resistant hypertension: a meta-analysis of randomized controlled trials. J Clin Hypertension. (2016) 18:153–8. 10.1111/jch.12639 26278919 PMC8031627

[93] Sanchez-de-la-Torre M Khalyfa A Sanchez-de-la-Torre A Martinez-Alonso M Martinez-Garcia MA Barcelo A. Precision medicine in patients with resistant hypertension and obstructive sleep apnea: blood pressure response to continuous positive airway pressure treatment. J Am Coll Cardiol. (2015) 66:1023–32. 10.1016/j.jacc.2015.06.1315 26314530

[94] Prejbisz A Kolodziejczyk-Kruk S Lenders JWM Januszewicz A Primary aldosteronism and obstructive sleep apnea: is this a bidirectional relationship? hormone and metabolic research = hormon- und Stoffwechselforschung = hormones et metabolisme. (2017) 49:969–76. 10.1055/s-0043-122887 29202496

[95] Warchol-Celinska E Prejbisz A Kadziela J Florczak E Januszewicz M Michalowska I. Renal denervation in resistant hypertension and obstructive sleep apnea: randomized proof-of-concept phase II trial. Hypertension. (2018) 72:381–90. 10.1161/HYPERTENSIONAHA.118.11180 29941516

[96] Shi L Chen SJ Ma MY Bao YP Han Y Wang YM. Sleep disturbances increase the risk of dementia: a systematic review and meta-analysis. Sleep Med Rev. (2018) 40:4–16. 10.1016/j.smrv.2017.06.010 28890168

[97] Beebe DW Groesz L Wells C Nichols A McGee K. The neuropsychological effects of obstructive sleep apnea: a meta-analysis of norm-referenced and case-controlled data. Sleep. (2003) 26:298–307. 10.1093/sleep/26.3.298 12749549

[98] Stranks EK Crowe SF. The cognitive effects of obstructive sleep apnea: an updated meta-analysis. Arch Clin Neuropsychol. (2016) 31:186–93. 10.1093/arclin/acv087 26743325

[99] Emamian F Khazaie H Tahmasian M Leschziner GD Morrell MJ Hsiung GY. The association between obstructive sleep apnea and alzheimer's disease: a meta-analysis perspective. Front Aging Neurosci. (2016) 8:78. 10.3389/fnagi.2016.00078 27148046 PMC4828426

[100] Lim DC Veasey SC. Neural injury in sleep apnea. Curr Neurol Neurosci Rep. (2010) 10:47–52. 10.1007/s11910-009-0078-6 20425226

[101] Varga AW Wohlleber ME Gimenez S Romero S Alonso JF Ducca EL. Reduced slow-wave sleep is associated with high cerebrospinal fluid abeta42 levels in cognitively normal elderly. Sleep. (2016) 39:2041–8. 10.5665/sleep.6240 27568802 PMC5070758

[102] Baril AA Carrier J Lafreniere A Warby S Poirier J Osorio RS. Biomarkers of dementia in obstructive sleep apnea. Sleep Med Rev. (2018) 42:139–48. 10.1016/j.smrv.2018.08.001 30241998 PMC8803351

[103] Baril AA Gagnon K Brayet P Montplaisir J Carrier J Soucy JP Obstructive sleep apnea during REM sleep and daytime cerebral functioning: a regional cerebral blood flow study using high-resolution SPECT. J Cerebral Blood Flow Metab. 2018:271678x18814106 10.1177/0271678X18814106 PMC7238367 30465610

[104] Duffy SL Lagopoulos J Terpening Z Lewis SJ Grunstein R Mowszowski L. Association of anterior cingulate glutathione with sleep apnea in older adults at-risk for dementia. Sleep. (2016) 39:899–906. 10.5665/sleep.5650 26856906 PMC4791623

[105] Quan SF Wright R Baldwin CM Kaemingk KL Goodwin JL Kuo TF. Obstructive sleep apnea-hypopnea and neurocognitive functioning in the sleep heart health study. Sleep Med. (2006) 7:498–507. 10.1016/j.sleep.2006.02.005 16815753

[106] Quan SF Chan CS Dement WC Gevins A Goodwin JL Gottlieb DJ. The association between obstructive sleep apnea and neurocognitive performance–the apnea positive pressure long-term efficacy study (APPLES). Sleep. (2011) 34:303–14b. 10.1093/sleep/34.3.303 21358847 PMC3041706

[107] Zimmerman ME Aloia MS. Sleep-disordered breathing and cognition in older adults. Curr Neurol Neurosci Rep. (2012) 12:537–46. 10.1007/s11910-012-0298-z 22752614 PMC3697017

[108] Del Brutto OH Mera RM Zambrano M Castillo PR. Relationship between obstructive sleep apnea and neuroimaging signatures of cerebral small vessel disease in community-dwelling older adults. the atahualpa project. Sleep Med. (2017) 37:10–2. 10.1016/j.sleep.2017.06.009 28899518

[109] Song TJ Park JH Choi KH Chang Y Moon J Kim JH. Moderate-to-severe obstructive sleep apnea is associated with cerebral small vessel disease. Sleep Med. (2017) 30:36–42. 10.1016/j.sleep.2016.03.006 28215260

[110] Rostanski SK Zimmerman ME Schupf N Manly JJ Westwood AJ Brickman AM. Sleep disordered breathing and white matter hyperintensities in community-dwelling elders. Sleep. (2016) 39:785–91. 10.5665/sleep.5628 27071695 PMC4791612

[111] Cross NE Memarian N Duffy SL Paquola C LaMonica H D'Rozario A. Structural brain correlates of obstructive sleep apnoea in older adults at risk for dementia. Eur Respir J. (2018) 52:1800740. 10.1183/13993003.00740-2018 29973356

[112] Nikodemova M Finn L Mignot E Salzieder N Peppard PE. Association of sleep disordered breathing and cognitive deficit in APOE epsilon4 carriers. Sleep. (2013) 36:873–80. 10.5665/sleep.2714 23729930 PMC3649829

[113] Fazekas F Enzinger C Ropele S Schmidt H Schmidt R Strasser-Fuchs S. The impact of our genes: consequences of the apolipoprotein E polymorphism in Alzheimer disease and multiple sclerosis. J Neurol Sci. (2006) 245:35–9. 10.1016/j.jns.2005.08.018 16631796

[114] Liguori C Mercuri NB Izzi F Romigi A Cordella A Sancesario G. Obstructive sleep apnea is associated with early but possibly modifiable alzheimer's disease biomarkers changes. Sleep. (2017) 40:zsx011. 10.1093/sleep/zsx011 28329084

[115] Bubu OM Pirraglia E Andrade AG Sharma RA Gimenez-Badia S Umasabor-Bubu OQ. Obstructive sleep apnea and longitudinal Alzheimer's disease biomarker changes. Sleep. (2019) 42:zsz048. 10.1093/sleep/zsz048 30794315 PMC6765111

[116] Osorio RS Ayappa I Mantua J Gumb T Varga A Mooney AM. Interaction between sleep-disordered breathing and apolipoprotein E genotype on cerebrospinal fluid biomarkers for Alzheimer's disease in cognitively normal elderly individuals. Neurobiol Aging. (2014) 35:1318–24. 10.1016/j.neurobiolaging.2013.12.030 24439479 PMC4022140

[117] Chowdhuri S Quan SF Almeida F Ayappa I Batool-Anwar S Budhiraja R. An official american thoracic society research statement: impact of mild obstructive sleep apnea in adults. Am J Respir Crit Care Med. (2016) 193:e37–54. 10.1164/rccm.201602-0361ST 27128710

[118] Holmes TH Kushida CA. Adherence to continuous positive airway pressure improves attention/psychomotor function and sleepiness: a bias-reduction method with further assessment of APPLES. Sleep Med. (2017) 37:130–4. 10.1016/j.sleep.2017.06.022 28899524 PMC5609486

[119] Cooke JR Ayalon L Palmer BW Loredo JS Corey-Bloom J Natarajan L. Sustained use of CPAP slows deterioration of cognition, sleep, and mood in patients with Alzheimer's disease and obstructive sleep apnea: a preliminary study. J Clin Sleep Med. (2009) 5:305–9. 19968005 PMC2725246

[120] Richards KC Gooneratne N Dicicco B Hanlon A Moelter S Onen F. CPAP adherence may slow 1-year cognitive decline in older adults with mild cognitive impairment and apnea. J Am Geriatrics Soc. (2019) 67:558–64. 10.1111/jgs.15758 30724333 PMC6402995

