# Supplementary material for: Hypertension and Cognitive Decline: Implications of Obstructive Sleep Apnea
Source: Front Cardiovasc Med. 2019 Jul 10;6:96. doi: 10.3389/fcvm.2019.00096 (PMC6636426; doi:10.3389/fcvm.2019.00096)
Supplement: Supplementary file 1 [file Table_1.pdf]

frontiers in Cardiovascular Medicine

SUPPLEMENTARY MATERIAL

doi: 10.3389/fcvm.2019.00096

Hypertension and Cognitive Decline: Implications of Obstructive Sleep Apnea

Meghna P. Mansukhani[1], Bhanu Prakash Kolla[1,2] and Virend K. Somers[3]*

[1] Center for Sleep Medicine, Mayo Clinic, Rochester, MN, United States, [2] Department of Psychiatry and Psychology, Mayo Clinic, Rochester, MN, United States, [3] Department of Cardiovascular Diseases, Mayo Clinic, Rochester, MN, United States

TABLE S1 | Summary of epidemiologic studies examining the association between midlife elevated BP and cognitive decline/dementia.

References	Population	Main findings
Farmer et al. (1)	Framingham study, N = 1,993, 27–87 years, US	Mean SBP and DBP were inversely associated with cognitive function 28 years later
Launer et al. (2)	Honolulu-Asia Aging study, N = 3,735 Japanese males living in Hawaii, US	After accounting for confounders, high SBP (but not DBP) was associated with increased risk of intermediate and poor cognitive function in late life (mean age 78 years)
Swan et al. (3)	Western Collaborative Group Study, N = 717, 39–59 years, US	Those with persistently high SBP starting in midlife were at increased risk of reduced verbal learning and memory function compared to those with normal SBP; those with low SBP had reduced psychomotor speed
Swan et al. (4)	National Heart, Lung, and Blood Institute (NHLBI) Twin Study, N = 392 male, 43–53 years, US	High midlife SBP predicted cognitive decline and magnetic resonance volumetric measures of brain atrophy in late life
Kilander et al. (5)	N = 999, mean age 50 years, Sweden	Elevated DBP was associated with greater cognitive decline 20 years later, especially in those untreated
Cerhan et al. (6)	Atherosclerosis risk in communities (ARIC) study, N = 13,840, 45–69 years, US, cross-sectional	Hypertension was associated with poorer performance on cognitive measures only in women
Tzouiro et al. (7)	N = 717, 59–71 years, France	Hypertension was associated with greater cognitive decline over 4 years, especially in those untreated
Launer et al. (8)	Honolulu-Asia Aging study, N = 3,703 Japanese American males, US	High SBP and DBP were independently associated with AD and vascular dementia in late life in those who were never treated with antihypertensive medication compared to those with normal SBP and DBP
Kilander et al. (9)	N = 463 males, 69–74 years, Sweden	Low DBP at age 50 years was associated with better performance on cognitive tests assessing subcortico-frontal function 20 years later in those without stroke
Knopman et al. (10)	ARIC study, N = 10963, 47–70 years, US	Hypertension was associated with greater decline in processing speed over 6 years
Pavlik et al. (11)	NHANES (National Health and Nutrition Examination Survey) study III, N = 3270, 30–59 years, US, cross-sectional	Hypertension and diabetes mellitus together were associated with worse working memory, processing speed and reaction time
Singh-Manoux et al. (12)	Whitehall II study, N = 5,838, mean age 44 years, UK	Elevated SBP was associated with worse memory and verbal fluency scores 12 years later, particularly in women
Wolf et al. (13)	Framingham Heart Study, N = 1,814, 40–69 years, US	Hypertension was associated with worse performance on measures of visual memory and executive functioning 12 years later
Knopman et al. (14)	ARIC study, N = 1,130, mean 59 years, 62% female, 52% Black, US, longitudinal study on vascular risk factors, APOE genotype and cognition	Hypertension in midlife was independently associated with decline in the word fluency test but not in other cognitive tests 14 years later
Shah et al. (15)	Honolulu-Asia Aging study, N = 667 Japanese American males, mean age 58 years, US	Plasma beta-amyloid related risk of AD was higher when midlife BP was higher
Power et al. (16)	Normative aging study, N = 758, mean age 37 years, US	Hypertension diagnosed at any point and greater duration of hypertension were associated with lower cognitive functioning 29 years later
Gottesman et al. (17)	ARIC study, N = 13,476, 48–67 years (58% completed follow up), 3 US communities	After accounting for confounders, high midlife BP and hypertension were associated with more cognitive decline on testing 20 years later (in Whites more than Blacks), this association was not seen with late life high BP
Gillett et al. (18)	Reasons for Geographic and Racial Differences in Stroke (REGARDS) study, N = 17,630, mean age 64 years, US	Hypertension was not associated with the development of cognitive decline over 40 months
Shang et al. (19)	N = 1,799, 40–85 years, China, cross-sectional	Elevated SBP and DBP were associated with cognitive decline in middle-aged but not older adults
Gottesman et al. (20)	ARIC-positron emission tomography study, N = 322, 45–64 years, 58% female, 43% Black, 3 US communities	After accounting for confounders, vascular risk factors including high BP were associated with increased amyloid deposition on PET scan 20 years later, this association was not seen with late life high BP
Gottesman et al. (21)	ARIC study, N = 15, 744, 44–66 years, 27% Black, 3 US communities	Pre-hypertension and hypertension were independently associated with incident dementia 25 years later

SBP, systolic blood pressure; DBP, diastolic blood pressure; AD, Alzheimer's dementia; APOE, Apolipoprotein E.

REFERENCES

1. Farmer ME, Kittner SJ, Abbott RD, Wolz MM, Wolf PA, White LR. Longitudinally measured blood pressure, antihypertensive medication use, and cognitive performance: the framingham study. *J Clin Epidemiol.* (1990) 43:475–80. doi: 10.1016/0895-4356(90)90136-D
2. Launer LJ, Masaki K, Petrovitch H, Foley D, Havlik RJ. The association between midlife blood pressure levels and late-life cognitive function. the honolulu-Asia aging study. *JAMA*. (1995) 274:1846–51. doi: 10.1001/jama.274.23.1846
3. Swan GE, Carmelli D, Larue A. Systolic blood pressure tracking over 25 to 30 years and cognitive performance in older adults. *Stroke.* (1998) 29:2334–40. doi: 10.1161/01.STR.29.11.2334

4. Swan GE, DeCarli C, Miller BL, Reed T, Wolf PA, Jack LM, et al. Association of midlife blood pressure to late-life cognitive decline and brain morphology. *Neurology.* (1998) 51:986–93. doi: 10.1212/WNL.51.4.986
5. Kilander L, Nyman H, Boberg M, Hansson L, Lithell H. Hypertension is related to cognitive impairment: a 20-year follow-up of 999 men. *Hypertension.* (1998) 31:780–6. doi: 10.1161/01.HYP.31.3.780
6. Cerhan JR, Folsom AR, Mortimer JA, Shahar E, Knopman DS, McGovern PG, et al. Correlates of cognitive function in middle-aged adults. atherosclerosis risk in communities (ARIC) study investigators. *Gerontology.* (1998) 44:95–105. doi: 10.1159/000021991
7. Tzourio C, Dufouil C, Ducimetiere P, Alperovitch A. Cognitive decline in individuals with high blood pressure: a longitudinal study in the elderly. EVA study group. epidemiology of vascular aging. *Neurology.* (1999) 53:1948–52. doi: 10.1212/WNL.53.9.1948
8. Launer LJ, Ross GW, Petrovitch H, Masaki K, Foley D, White LR, et al. Midlife blood pressure and dementia: the Honolulu-Asia aging study. *Neurobiol Aging.* (2000) 21:49–55. doi: 10.1016/S0197-4580(00)00096-8
9. Kilander L, Nyman H, Boberg M, Lithell H. The association between low diastolic blood pressure in middle age and cognitive function in old age. a population-based study. *Age Age.* (2000) 29:243–8. doi: 10.1093/ageing/29.3.243
10. Knopman D, Boland LL, Mosley T, Howard G, Liao D, Szklo M, et al. Cardiovascular risk factors and cognitive decline in middle-aged adults. *Neurology.* (2001) 56:42–8. doi: 10.1212/WNL.56.1.42
11. Pavlik VN, Hyman DJ, Doody R. Cardiovascular risk factors and cognitive function in adults 30–59 years of age (NHANES III). *Neuroepidemiology.* (2005) 24:42–50. doi: 10.1159/000081049
12. Singh-Manoux A, Marmot M. High blood pressure was associated with cognitive function in middle-age in the Whitehall II study. *J Clin Epidemiol.* (2005) 58:1308–15. doi: 10.1016/j.jclinepi.2005.03.016
13. Wolf PA, Beiser A, Elias MF, Au R, Vasan RS, Seshadri S. Relation of obesity to cognitive function: importance of central obesity and synergistic influence of concomitant hypertension. the framingham heart study. *Curr Alzheimer Res.* (2007) 4:111–6. doi: 10.2174/156720507780362263
14. Knopman DS, Mosley TH, Catellier DJ, Coker LH. Fourteen-year longitudinal study of vascular risk factors, APOE genotype, and cognition: the ARIC MRI study. Alzheimer's & dementia. *J Alzheimer's Assoc.* (2009) 5:207–14. doi: 10.1016/j.jalz.2009.01.027
15. Shah NS, Vidal JS, Masaki K, Petrovitch H, Ross GW, Tilley C, et al. Midlife blood pressure, plasma beta-amyloid, and the risk for Alzheimer disease: the Honolulu Asia aging study. *Hypertension.* (2012) 59:780–6. doi: 10.1161/HYPERTENSIONAHA.111.178962
16. Power MC, Tchetgen EJ, Sparrow D, Schwartz J, Weisskopf MG. Blood pressure and cognition:factors that may account for their inconsistent association. *Epidemiology (Cambridge, Mass).* (2013) 24:886–93. doi: 10.1097/EDE.0b013e3182a7121c
17. Gottesman RF, Schneider AL, Albert M, Alonso A, Bandeen-Roche K, Coker L, et al. Midlife hypertension and 20-year cognitive change: the atherosclerosis risk in communities neurocognitive study. *JAMA Neurol.* (2014) 71:1218–27. doi: 10.1001/jamaneurol.2014.1646
18. Gillett SR, Thacker EL, Letter AJ, McClure LA, Wadley VG, Unverzagt FW, et al. Correlates of incident cognitive impairment in the reasons for geographic and racial differences in stroke (REGARDS) study. *Clin Neuropsychol.* (2015) 29:466–86. doi: 10.1080/13854046.2015.1042524
19. Shang S, Li P, Deng M, Jiang Y, Chen C, Qu Q. The age-dependent relationship between blood pressure and cognitive impairment: a cross-sectional study in a rural area of Xi'an, China. *PloS ONE.* (2016) 11:e0159485. doi: 10.1371/journal.pone.0159485
20. Gottesman RF, Schneider AL, Zhou Y, Coresh J, Green E, Gupta N, et al. Association between midlife vascular risk factors and estimated brain amyloid deposition. *JAMA.* (2017) 317:1443–50. doi: 10.1001/jama.2017.3090
21. Gottesman RF, Albert MS, Alonso A, Coker LH, Coresh J, Davis SM, et al. Associations between midlife vascular risk factors and 25-year incident dementia in the atherosclerosis risk in communities (ARIC) cohort. *JAMA Neurol.* (2017) 74:1246–54. doi: 10.1001/jamaneurol.2017.1658
